# Over-expression of Topoisomerase II Enhances Salt Stress Tolerance in Tobacco

**DOI:** 10.3389/fpls.2016.01280

**Published:** 2016-08-31

**Authors:** Riffat John, Uma Ganeshan, Badri N. Singh, Tanushri Kaul, Malireddy K. Reddy, Sudhir K. Sopory, Manchikatla V. Rajam

**Affiliations:** ^1^Plant Molecular Biology Laboratory, Department of Botany, University of KashmirSrinagar, India; ^2^Plant Polyamine, Transgenic and RNAi Laboratory, Department of Genetics, University of Delhi South CampusNew Delhi, India; ^3^Plant Biology, International Centre for Genetic Engineering and BiotechnologyNew Delhi, India

**Keywords:** tobacco, topoisomerase II, transgenic plants, salinity stress, stress tolerance, glycine betaine

## Abstract

Topoisomerases are unique enzymes having an ability to remove or add DNA supercoils and untangle the snarled DNA. They can cut, shuffle, and religate DNA strands and remove the torsional stress during DNA replication, transcription or recombination events. In the present study, we over-expressed topoisomerase II (TopoII) in tobacco (*Nicotiana tabaccum*) and examined its role in growth and development as well as salt (NaCl) stress tolerance. Several putative transgenic plants were generated and the transgene integration and expression was confirmed by PCR and Southern blot analyses, and RT-PCR analysis respectively. Percent seed germination, shoot growth, and chlorophyll content revealed that transgenic lines over-expressing the *NtTopoII*α-1 gene exhibited enhanced tolerance to salt (150 and 200 mM NaCl) stress. Moreover, over-expression of *TopoII* lead to the elevation in proline and glycine betaine levels in response to both concentrations of NaCl as compared to wild-type. In response to NaCl stress, *TopoII* over-expressing lines showed reduced lipid peroxidation derived malondialdehyde (MDA) generation. These results suggest that TopoII plays a pivotal role in salt stress tolerance in plants.

## Introduction

Unwinding of the DNA double helix is either transient as in transcription and recombination or stable as during DNA replication (Champoux, 2001). DNA being negatively supercoiled, the strand separation by helicases first relaxes DNA but further duplex melting leads to positively supercoiled strands that impede the action of helicases (Capranico et al., 2010). Topoisomerases ubiquitously present in both prokaryotes and eukaryotes play a crucial role in untangling and relaxing the interwound DNA supercoiled duplex, thereby mitigating the topological stress (Vos et al., 2011; Ashour et al., 2015). Topoisomerases remarkably cut, shuffle and rejoin DNA strands and are pre-requisite for proper chromosomal organization and segregation (Nitiss, 2009).

DNA topoisomerase was first isolated from *Escherichia coli* by James Wang in 1971 and was named as ω protein now known as DNA topoisomerase I (TopoI; Wang, 1971). Toposiomerases are extraordinarily diverse and quiet complex in their mode of action and divided into two categories: Type I and type II (Singh et al., 2003, 2004; Vrielynck et al., 2016). Type I topoisomerases are ATP independent-monomeric proteins which nick and seal only one strand of DNA thus changing the linking number of supercoiled DNA by steps of one, whereas the type II topoisomerases are ATP-dependent involved in nicking both strands of DNA to create a staggered double-strand break and thereby changing the linking number by steps of two (Pyke et al., 1989; Hsieh, 1990).

Topoisomerase II (TopoII), a dimeric enzyme, is located at the base of the chromosomal DNA loops that changes the topology of DNA by nicking two strands of DNA to form a gate, then directing another DNA molecule to pass through the gate and finally resealing the nicks to close the gate (Tammaro et al., 2016). In addition, TopoII plays a role in removing positive superturns ahead of replication forks and moving transcription complexes in archaea and bacteria, since the TopoIA present in these two domains cannot relax positive superturns (Gadelle et al., 2003). All eukaryotic type II topoisomerases characterized till now show similarity in their primary structure comprising of 1429–1530 amino acid residues with a predictable molecular mass of 160–170 kDa (Wang, 2002; Corbett and Berger, 2004). In the recent past, there has been a surge in biochemical and biological aspects of topoII and various processes underlying its functions and reactions have been established (Nitiss, 2009; Blattner, 2016) but studies are mostly restricted to animal system. TopoII has been extensively studies in animals not only with reference to change in DNA topology but with respect to a large number of topoisomerase-targeted drugs that have been identified possessing antimicrobial and anticancerous activities (Kathiravan et al., 2013; Ashour et al., 2014). Most of these drugs interfer with the processing of the DNA break, causing stabilization of transient covalent DNA–DNA topoisomerase complex into a form that is harmful for the cell (Gadelle et al., 2003).

Abiotic stress tolerance is a multigenic trait. Although large number of genes have been identified in plants in response to abiotic stress but unraveling the relationship between DNA replication enzymes and abiotic stress is crucial for developing abiotic stress tolerant transgenic crops (Hettiarachchi et al., 2005). Earlier, we have isolated and characterized *TopoII* from *Nicotiana tabacum*. We proposed that *TopoII* under GAL1 promoter functionally complements a temperature-sensitive topoisomerase II^ts^ yeast mutant (Singh et al., 2003) and its transcript levels get up-regulated in response to cold and salinity stress in tobacco and pea (Hettiarachchi et al., 2005). Here, we report that over-expression of *TopoII* in *N. tabaccum* leads to enhanced salinity stress tolerance.

## Materials and methods

### Transformation of *Agrobacterium*

Recombinant plasmid pBI121- NtTopIIα-1 was transferred into *Agrobacterium* by freeze-thaw method. Competent cells were prepared by growing *Agrobacterium tumefaciens* (LBA4404) in 50 ml YEM medium (0.04% yeast extract, 1% mannitol, 0.01% NaCl, 0.02% MgSO_4_.7H_2_O and 0.05% K_2_HPO_4_) at 28°C with vigorous shaking until 0.5 OD_600_ was achieved. Later, the culture was chilled on ice and then centrifuged at 3000 x g for 5 min at 4°C. After suspending the pellet in 1 ml of ice cold CaCl_2_ (20 mM), 0.1 ml aliquots were dispensed in pre-chilled Eppendorf tubes and stored at −80°C.

### *Agrobacterium*-mediated tobacco transformation

Tobacco seeds (*N. tabaccum*, cv Petit Havana) were sterilized by soaking in a bleach solution (2% w/v) for 15–20 min followed by three washes in sterile water. Seeds were germinated on RM (rooting medium) (Macro nutrients: 1.9 g KNO_3_, 0.37 g MgSO_4_.7H_2_O, 0.44 g CaCl_2_.2H_2_O, 0.17 g KH_2_PO_4_, 1.65 g (NH_4_)_2_NO_3_; Micro nutrients: 1.6 mg MnSO_4_.2H_2_O, 0.62 mg H_3_BO_3_, 0.86 mg ZnSO_4_.7H_2_O, 0.83 mg KI, 0.025 mg Na_2_MoO_4_.2H_2_O, 0.0025 mg CuSO_4_.5H_2_O, 0.0025 mg CoCl_2_.6H_2_O; 50 mg Fe-EDTA, 30 g Sucrose and 6% agar per liter, pH 5.8) and kept under moderate light, temperature, and low humidity conditions till plants with healthy leaves were produced. Uniform sized leaves were harvested and leaf discs were prepared by cutting the leaves into small squares (1 cm^2^). The leaf discs were then immersed in *Agrobacterium* cells [1:10 dilution of overnight grown culture in liquid RMOP (MS containing 1 mg/L benzyladenine (BAP), 3% sucrose, and 7 g/l phytoagar)] for 5 min harboring recombinant vector pBI121-NtTopIIα-1 for *TopoII* gene under the control of CaMV35S promoter and nopaline synthase terminator, and *npt*-II gene as plant selection marker. Leaf discs were taken out, blot dried and placed upside down on the RMOP culture plates. The explants were allowed to co-culture with *Agrobacterium* for 2–3 days. Immediately after co-cultivation, explants were transferred to RMOP medium (RM supplemented with 1 mg thiamine, 1 mg NAA, 1 mg BAP, and 100 mg inositol) containing 200 μg kanamycin and 500 μg/ml carbenicillin. After 3–4 weeks, shoots with a defined stem were removed carefully from explants and placed on MS rooting medium. Following rooting, plantlets were removed from culture jars and rinsed in water and planted in pots containing vermiculite. Pots were covered with plastic bags (with pin holes) and closed tightly to retain humidity. After 7–10 days, the bags were removed in order to reduce the humidity gradually until plants became acclimatized to the ambient humidity. Thereafter, the plants were transferred to soil and grown under green-house condition to allow blooming and seed setting.

### Screening of putative tobacco transgenic plants by PCR analysis

Genomic DNA was isolated from leaf tissue of tobacco transformants and untransformed plants by CTAB method (Doyle and Doyle, 1990). The putative transgenic plants were analyzed by PCR for the integration of *Topo*II and *npt*-II transgenes. The conditions for PCR were 40 cycles of denaturation at 94°C for 1 min, annealing for 1 min at 52°C, annealing for 1 min at 54°C for *npt*-II gene, while 65°C for *TopoII* gene and synthesis at 72°C for 2 min, and finally the extension step of 10 min at 72°C. The PCR products were checked on 1% agarose gel with the ladder. The primer pairs for the amplification of a 675 kb fragment of *npt*-II gene were -5′CCCATGAAAAAG CCTGGACTCACCGCG3′ and 5′ GCAGGCTCC CGTTTCCTTATCGAT3′. Primer pairs specific for the amplification of a 960 kb fragment for *TopoII* gene were 5′ CTGCCCAAAGAA GAAACAGG 3′- and 5′ AACCAAAAGCCA GAGGAGGTC 3′.

### Southern analysis

Genomic DNA (10 mg) from PCR positive transgenic plants and wild-type plants of tobacco was restricted with *Xho*I for checking the transgene integration and copy number and probed with *npt*-II gene. Southern blots were prepared by standard procedure (Sambrook et al., 1989) using Hybond-N Nylon membrane (Pharmacia). The *npt*-II gene probe was prepared using ^32^P-labeled dCTP by nick translation as per the manufacture's guidelines (Gibco-BRL). Hybridization was carried out for 18–24 h at 65°C. The membrane was washed and then exposed to X-ray film (XK-5Kodak film).

### RNA extraction and semi-quantitative RT-PCR analysis

Total RNA was isolated as per the protocol described by Chomczynski and Sacchi (1987). RT-PCR was done to check the level of transcription of the transformed lines of tobacco. Total RNA at the concentration of 1.5 mg (treated with RNase-free DNase) was used as a template and was mixed with 1X buffer, 1X Q solution, 400 mM of dNTPmix, 1.2 mM primers, 5 U of RNase inhibitor, and 2 ml of enzyme mix (Reverse Transcriptase and Taq Polymerase). The reaction volume was made to 25 ml and incubated at 50°C for 30 min. After reverse transcription, the reaction mixture was heated to 95°C for 15 min (to activate HotStart Taq DNA polymerase and to simultaneously inactivate the reverse transcriptase), followed by 30 cycles of 1 min denaturation at 94°C, primer annealing at 65°C for 1 min, extension at 72°C for 1 min and final extension for 10 min. The PCR products were analyzed on 1% agarose gel. Densitometric analysis of protein bands was performed using the public software ImageJ 1.41o developed by Wayne Rasband at the National Institutes of Health.

### Transgene segregation

One hundred T_1_ seeds obtained from primary transgenic plants of *TopoII* were used for the segregation of the transgenes. The surface sterilized seeds were inoculated on to MS basal medium supplemented with 200 mg/l kanamycin and incubated at 26°C ± 1 and 16 h photoperiod. The germination was observed up to 10 days and the percentage germination was scored for segregation analysis after 1 month. The experiment was repeated thrice.

### Seed germination, seedling growth, leaf disc assay, and chlorophyll content

Fifty seeds from control and T_1_ transgenic lines were surface-sterilized and inoculated on to MS basal medium supplemented with 150 mM and 200 mM NaCl for salt tolerance assays. Tolerance was based on percent seed germination. Kanamycin resistant 12 T_1_ seedlings with a well-developed root system were grown in culture bottles containing vermiculite: Soil (1:1) mix. The medium was supplemented with 200 mM NaCl for salt stress for 10–15 days. The tolerance was based on the survival and growth of the seedlings under stressed condition. Healthy leaves from wild-type and T_1_ transgenic tobacco plants (of similar age) were washed in deionized water, and 12 leaf discs of 1 cm diameter were punched out with cork borer and floated in a solution of NaCl (150 mM and 200 mM) or sterile distilled water (which served as experimental control) in petridishes. The chlorophyll content was measured as per Arnon (1949). The experiments were repeated thrice with similar samples.

### Estimation of proline, lipid peroxidation, and glycine betaine content

The kanamycin resistant T_1_ seedlings were grown in pots with vermiculite and soil mix (1:1) under controlled growth conditions. The seedlings were watered with 1/10 MS medium with two different concentrations of NaCl, i.e., 150 mM and 200 mM. Plants were grown for a period of 1 month and harvested for different assays. Free proline in leaf tissue was assayed following the protocol of Bates et al. (1973). The level of lipid peroxidation was measured by estimating MDA, using thiobarbituric acid (TBA) as the reactive material following the method of Heath and Packer (1968). Glycine betaine content in fresh leaf samples was measured according to Grieve and Grattan (1983). The absorbance was measured by spectrophotometer at 365 nm while glycine betaine (50–200 mg/ml) prepared in 1N H_2_SO_4_ was used as control. The experiments were done in triplicates and repeated thrice.

### Statistical analyses

Data were analyzed by SPSS 16.0 (SPSS Corp., Chicago, IL, USA) and subjected to statistical analysis of variance (ANOVA) with lines (One way ANOVA) and lines and treatments (Two-way ANOVA) as factors, followed by Tukey's test and least significant difference (LSD) test. A *P*-value less than 0.05 were considered statistically significant. All experiments were carried out three times.

## Results

### Tobacco transformation, regeneration, and morphology

**Figure 1:**
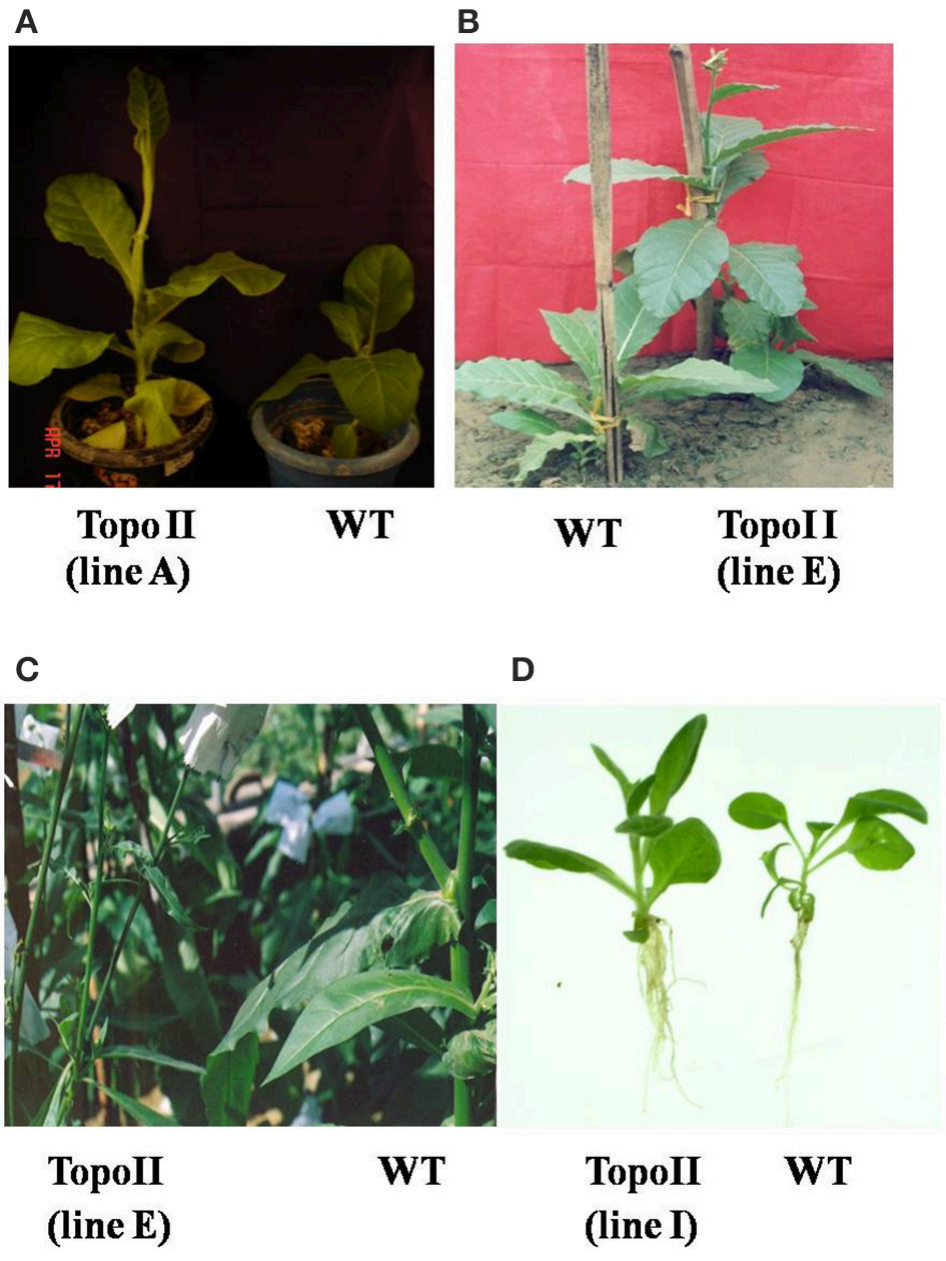
**Morphological changes in *TopoII* transgenic tobacco lines at different growth stages in comparison to WT**. **(A,B)** Pre-flowering stage; **(C)** Post-flowering stage and **(D)** Longer and profuse root growth in transgenic lines as compared to WT.

The co-cultivated explants with *TopoII* gene construct showed shoot regeneration after 8–13 days of culture on the selection medium. The transformation frequency, calculated as number of explants surviving and showing shoot regeneration on selection medium upon the total number of explants co-cultivated, was in the range of 24–37%. We observed that *TopoII* putative transgenic plants were morphologically different from control plants and showed early flowering and seed setting by 2–3 weeks. The *TopoII* expressing T_0_ and T_1_ transgenic lines had longer stems and robust root system as compared to the wild-type (WT) plants. Moreover, transgenics had thinner stem (reduced girth) and longer internodes. We observed about 12% increase in stem height and 11–13% internode length in tobacco transgenic plants. Interestingly stem thickness was reduced from 1.98 cm in control to 1.73 cm in transgenic lines (Figure 1, Table 1).

**Table 1:** **Morphological changes in tobacco due to over-expression of *TopoII* gene**.

**Transgenic line**	**Morphological parameters**		
**Line**	**Stem height (cm)**	**Stem thickness (cm)**	**Internode length (cm)**
WT	55.21 ± 2.12	1.98 ± 0.08	0.25 ± 0.01
A line	75.54 ± 3.43	1.69 ± 0.03	0.36 ± 0.01
D line	77.24 ± 4.27	1.72 ± 0.02	0.38 ± 0.03
E line	76.56 ± 4.88	1.74 ± 0.05	0.37 ± 0.02
F line	77.38 ± 3.13	1.80 ± 0.03	0.38 ± 0.01
I line	40.51 ± 2.51^*^	2.12 ± 0.01^*^	0.20 ± 0.01^*^

*Data are the mean and standard error, based on 12 T_1_ progeny of TopoII expressing transgenic lines*.

* *Significant at 5%. WT, wild-type*.

### Transgene integration and expression

**Figure 2:**
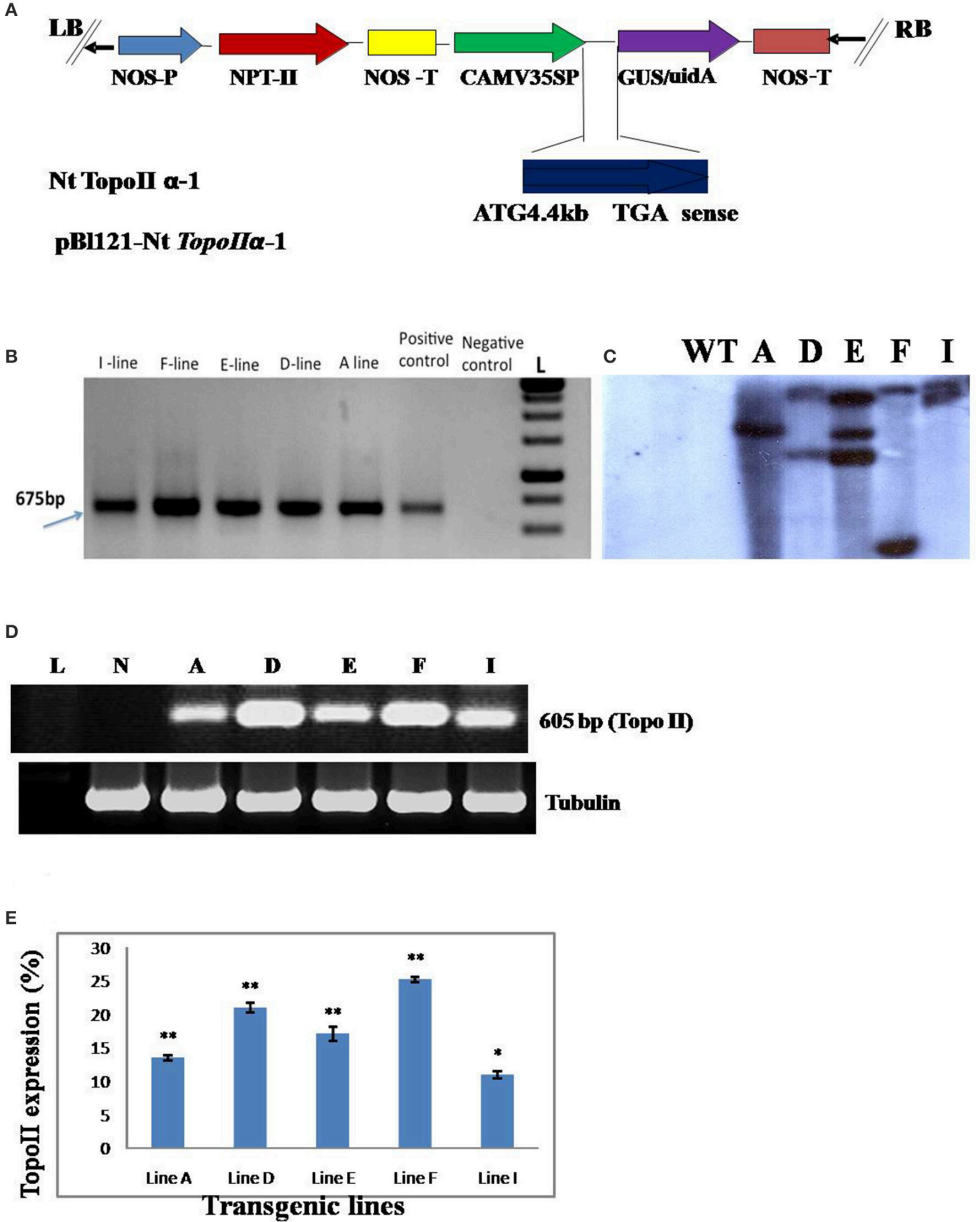
**(A)** T-DNA map of NtTopoIIα-1 construct used for tobacco transformation. NOS P, nopaline synthase promoter; NPT-II, neomycin phosphotransferase II; NOS T, nopaline synthase terminator; CaMV P, Cauliflower mosaic virus 35S promoter; GUS uidA, β-glucaronidase; **(B)** Genomic DNA PCR analysis of untransformed control and putative *TopoII* transgenic tobacco plants (A–I lines) using *npt*-II gene-specific primers. L, DNA Ladder; Positive control, Plasmid DNA; Negative control, DNA from untransformed tobacco plant; **(C)** Southern blot analysis of transgenic and wild-type (WT) tobacco plants determining the T-DNA copy number. Genomic DNA was digested with *Xho*I, separated by electrophoresis on a 1% agarose gel and transferred onto a nylon membrane. The transferred DNA was hybridized with the *npt*-II gene probe labeled with α-32P dCTP. UT, DNA from the untransformed tobacco plant, A–I, transgenic lines; **(D)** Transgene expression (transcript) levels quantified by RT-PCR. Tubulin was used as an internal control. L, DNA ladder; N, Blank; A–I, transgenic lines; **(E)** The histograms represent the average of gene expression measured with the ImageJ, densitometry software. Values are the means of 3 replicates with the corresponding standard error. Bars labeled with asterisks show significant differences from that of WT at ^*^P < 0.05 or ^**^P < 0.01 by *t*-test.

PCR analysis of *TopoII* expressing transgenic tobacco plants with screening primers specific to *npt*-II gene confirmed the transgene integration. The PCR positive plants were analyzed by Southern hybridization for revealing the copy number of transgene. Genomic DNA of *TopoII* transgenics was restricted with *Eco*RI and *Bam*HI separately and probed with *GUS* gene. The transgenic plants had single and multiple copy transgene insertionss. The transgene expression was analyzed in different transgenic lines by semi-quantitative RT-PCR using *TopoII* gene-specific primers and they exhibited variable expression of transgene in different lines (Figure 2).

### Segregation of the transgene

The T_1_ seeds obtained from primary transgenic plants were analyzed for the segregation of the transgene on solid MS basal medium supplemented with 200 mg/l kanamycin. The transgenic seeds germinated within 10 days of inoculation and showed segregation for the transgene on kanamycin-amended medium. The control seeds failed to germinate on kanamycin containing medium. The transgenic lines having a single copy of the transgene segregated in 3:1 ratio, while other lines containing multiple transgene integrations showed deviation from this ratio (data not shown).

### Salt stress response of transgenic lines

**Figure 3:**
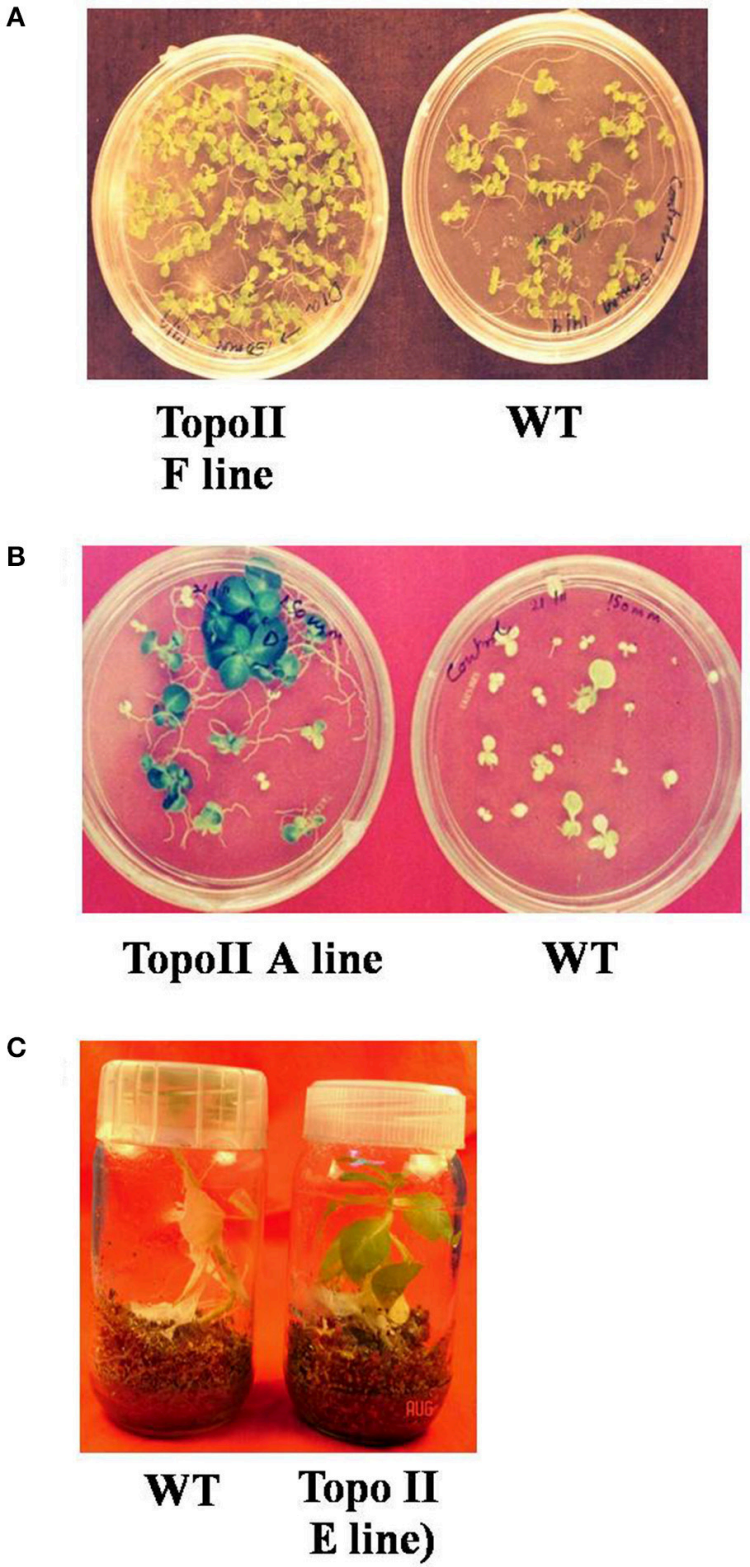
**Enhanced tolerance to different salt concentrations in *TopoII* over-expressing tobacco transgenic lines as compared to WT**. **(A)** Germination on MS medium supplemented with 150 mM; **(B)** Germination on MS medium supplemented with 200 mM NaCl and **(C)** Growth on vermiculite: Soil with 200 mM NaCl.

The seeds of T_1_ generation of transgenic *TopoII* tobacco lines obtained from self-pollinated primary transformants were checked for salt tolerance on the basis of seed germination, bleaching of leaf discs, and chlorophyll content under salt stress under both *in vitro* and *in vivo* growth conditions. We examined the effect of salt stress on germination of seeds from WT and transgenic lines. The surface-sterilized transgenic as well as WT seeds were inoculated on the solid MS basal medium containing 150 mM or 200 mM NaCl. The seeds from WT exhibited about 50% germination but *TopoII* transgenic lines showed 100% seed germination at 150 mM NaCl. At 200 mM NaCl, most of the seeds from transgenic lines germinated but the seeds from WT plants could not germinate under salt stress (Figures 3A,B, Table 2). The transgenic plants of tobacco, growing in vermiculite:sand supplemented with 200 mM for 25 days survived and showed better growth, but WT plants could not survive (Figure 3C).

**Table 2:** **Germination of wild-type and T_1_ transgenic seeds of tobacco on MS basal medium amended with 150 and 200 mM NaCl for salt stress tolerance**.

**Conc. of NaCl used (mM)**	**Total no. of seeds inoculated**	**No. of seeds germinated**					
		**WT**	**A line**	**D line**	**E line**	**F line**	**I line**
150	50	23	45	48	48	46	49
200	50	0	40	43	42	41	45

*Data are average of percent seed germination in control and TopoII transgenic tobacco lines. The experiment was repeated thrice with fifty T_1_ seeds of TopoII expressing transgenic lines in each experiment*.

**Figure 4:**
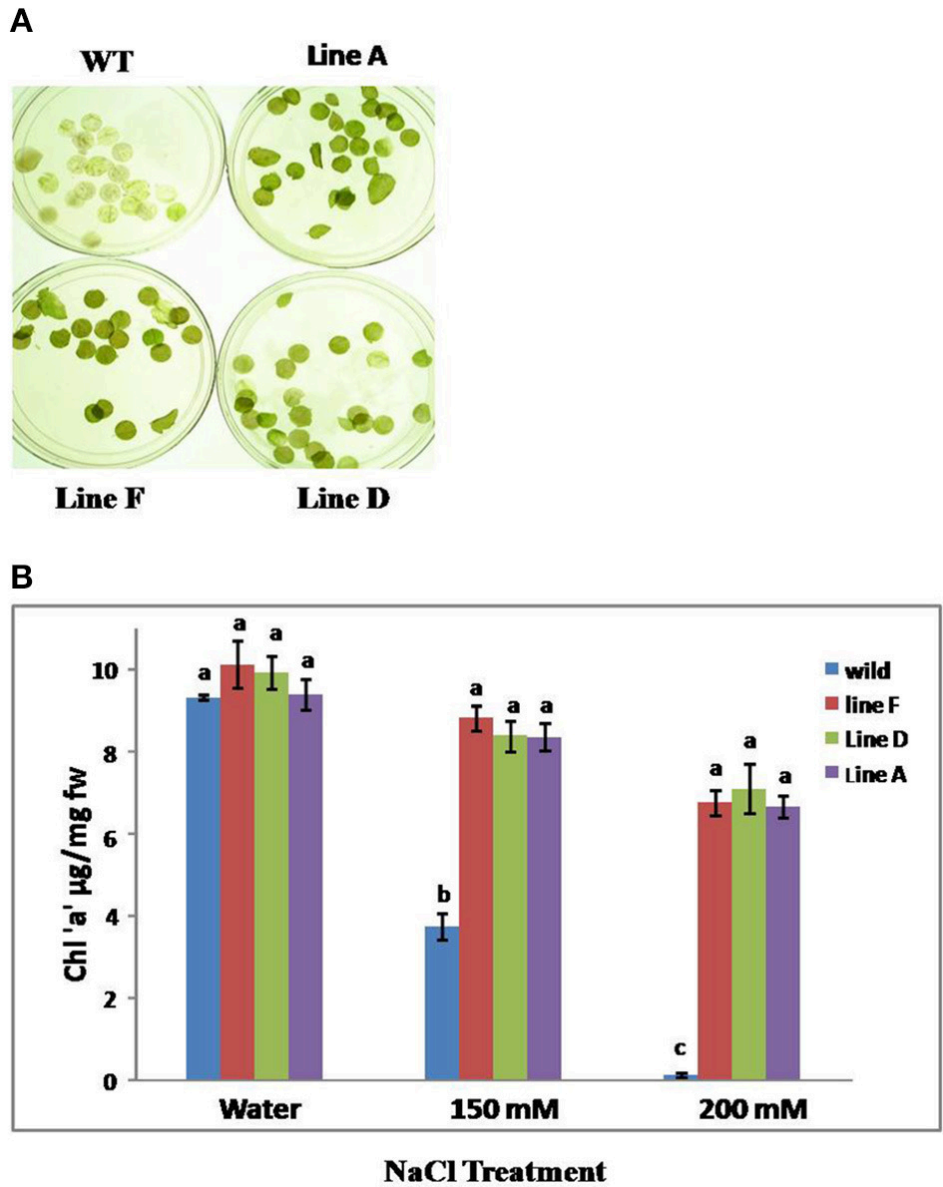
**(A)** Leaf disc assay for chlorophyll loss in WT and transgenic lines after 150 mM NaCl treatment and **(B)** Chlorophyll estimation after leaf disc assay with WT and *TopoII* over-expressing tobacco transgenic lines under salinity stress condition. Bars represent means and standard errors (*n* = 3) and those noted with same letters are not significantly different at *P* < 0.05 by Tukey's test.

The incubation of the leaf discs from WT and *TopoII* transgenic lines in 150 and 200 mM NaCl revealed an early bleaching of WT leaf discs as compared to transgenic lines. *TopoII* over-expressing lines of tobacco retained chlorophyll pigment at both 150 and 200 mM NaCl to a significant level as compared to control (Figures 4A,B).

### Over-expression of *Topoii* leads to the elevation in proline and glycine betaine levels and reduction in lipid peroxidation

**Figure 5:**
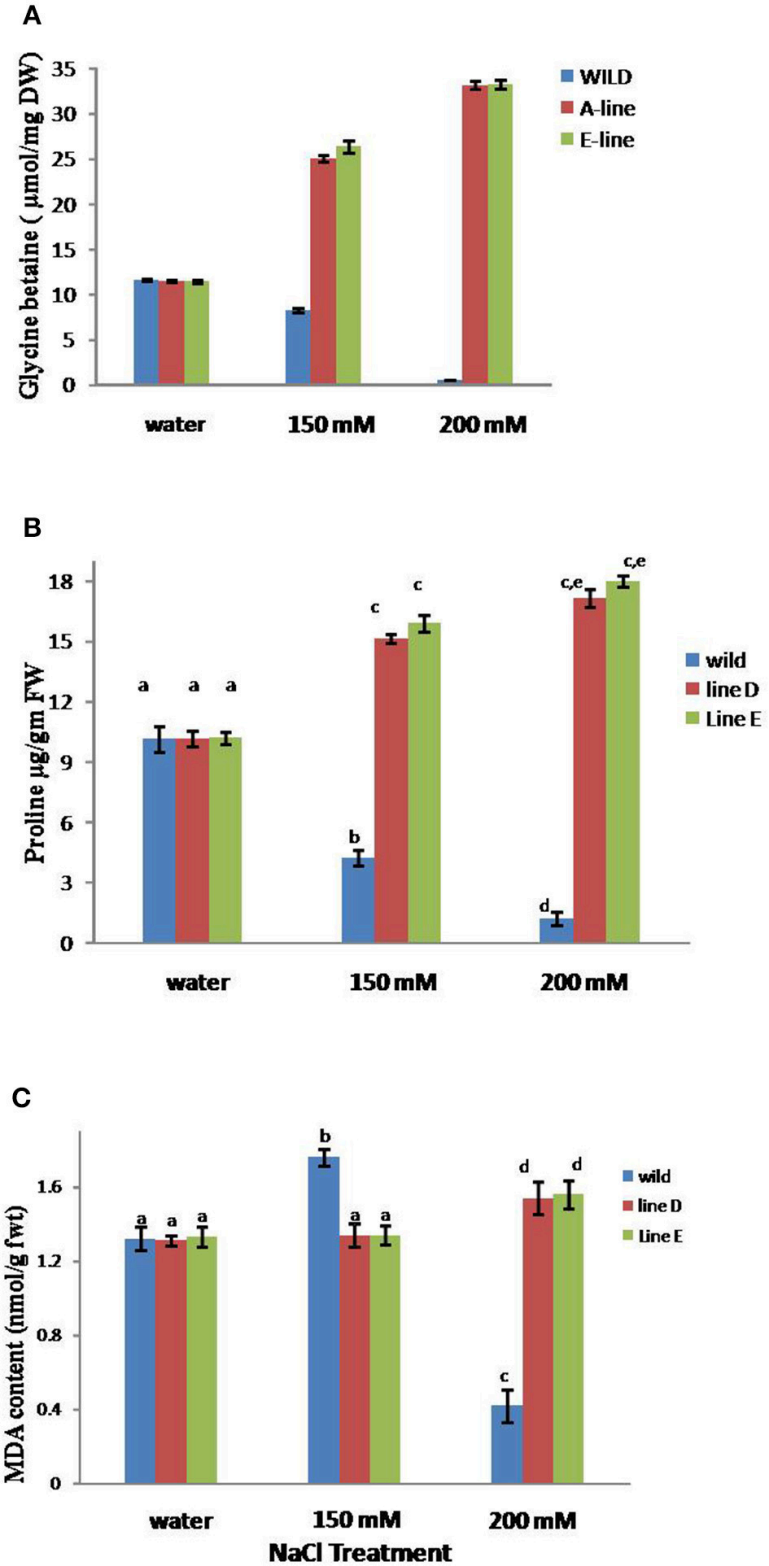
**Content of glycine betaine (A), proline (B), and MDA (C) in *TopoII* over-expressing tobacco and WT lines after treatment with 150 and 200 mM NaCl**. Bars represent means and standard errors (*n* = 3) and those noted with same letters are not significantly different at *P* < 0.05 by Tukey's test.

A significant increase in proline as well as glycine betaine content was recorded in transgenic lines under 150 and 200 mM salt stress. This suggests that up-regulation of topoisomrerase II activity leads to the stress tolerance by inducing proline and glycine betaine accumulation, while reducing the lipid peroxidation which was evident by reduced MDA content in the transgenic tobacco plants as compared to WT (Figure 5).

## Discussion

Apart from the role of topoisomerases in resolving problems associated with topological strains, they are being actively studied for their role as target for anticancerous drugs (Vann et al., 2016). In plants, most of the work on topoisomerases is focused on their isolation, purification and characterization (Badaracco et al., 1983) but their role in conferring abiotic stress tolerance in plants has not been explored.

In order to study the role of TopoII during salt stress in plants, it was over-expressed in tobacco. The transgenic tobacco plants over-expressing *NtTopoI*α showed altered plant morphology with longer stem and internode length but reduced girth as compared to WT tobacco plants. Such results corroborate with the observation by Takahashi et al. (2002), who reported that TOP1α mutation in *Arabidopsis* leads to the abnormal morphological pattern such as changes in number of floral primodia and internode length. TopoII has also been implicated in root stem cell niche maintainence in *Arabidopsis thalliana* (Yu et al., 2016). Our studies show that *TopoII* over-expression alters the morphology of the plants and these results suggest that topoisomerases play a critical role in regulating the developmental pattern in plants. Yin et al. (2002) also observed that mutation in *TopoVI* and *TopoVIB* homologs leads to pleiotropic dwarf phenotypes in *Arabidopsis*. Recently, we also reported that *TopoI* silencing in tobacco leads to the developmental abnormalities (Singh et al., 2015).

The leaf disc assay showed that tobacco plants with *TopoII* over-expression exhibited lesser loss of chlorophyll as compared to WT after putting them in 150 and 200 mM NaCl. Although the *TopoII* transgenic plants showed retention of chlorophyll but leaf discs from the WT showed complete bleaching. The decrease in chlorophyll content may be due to an increase of chlorophyll degradation as chlorophyllase activity under high salt concentration as seen in sunflower leaves (Santos, 2004). Also, Toposomerases may have role in chloroplast DNA replication as Marrison and Leech (1992) observed by localizing TopoII in chloroplast of wheat leaf that TopoII is associated with effective replication of chloroplast DNA.

We evaluated the *TopoII* expressing tobacco plants for biochemical parameters associated with NaCl stress such as the accumulation of proline, glycine betaine, and MDA content. A significant increase in the proline levels in NaCl tolerant transgenic lines was observed in comparison to WT and increase being higher when treated with 200 mM as compared to 150 mM NaCl. The proline content significantly elevated by 56 and 60% in transgenic lines A and E respectively. Higher free proline levels in response to salinity have been studied in different plants and possible roles of proline has been cited such as an osmolyte, stabilizing proteins, regulating cytosolic pH, and scavenging of hydroxyl radicals (Koca et al., 2007; Hazman et al., 2015; Dar et al., 2016). Glycine betaine levels also elevated in response to salt stress in transgenic lines by approximately 125% in both the transgenic lines when compared with WT. Various studies done at physiological level as well as genetics have verified that the levels of accumulated glycine betaine are proportional to the degree of salt tolerance (Sakamoto and Murata, 2002; Lai et al., 2014; Choi et al., 2016). Moreover, an exogenous supply of glycine betaine also increases the salt tolerance of some plants that are otherwise unable to accumulate glycine betaine (Hasanuzzaman et al., 2014). Glycine betaine effectively stabilizes the quaternary structures of enzymes and complex proteins, and maintains the highly ordered state of membranes under salinity stress (Chen and Murata, 2008). Also, glycine betaine is part of different signaling pathway which leads to salt stress tolerance via the activation of different stress related genes (Türkan and Demiral, 2009). Salt stress is known to cause lipid peroxidation, which has often been used as an indicator of salt-induced oxidative damage in membranes (Hernández and Almansa, 2002). On the contrary, we observed that MDA content was unaffected in salt stressed leaves of *TopoII* expressing transgenic lines in comparison to leaves of WT, which showed significant enhancement in response to NaCl (150 mM). In response to 200 mM NaCl, MDA levels were slightly higher in transgenic lines in comparison to 150 mM NaCl, but drastically reduced in WT. Results indicated that the over-expression of *TopoII* gene conferred enhanced tolerance to salt induced oxidative stress. Topoisomerases play a role in integration of multiple ROS signals released by plants in response to environmental stress (Šimková et al., 2012).

## Conclusion

Our study reveals that *NtTopoII1*-α over-expression in tobacco confers salt stress tolerance to the transformed lines as compared to wild-type plants. *TopoII* over-expression changed the morphology of the transgenic plants and improved the seed germination on salt supplemented medium. The transgenic lines also displayed higher levels of chlorophyll, proline, and glycine betaine under NaCl salt stress. Plants utilize different strategies to efficacously combat the adverse environmental conditions (e.g., salinity, drought, extreme temperatures, heavy-metal stress, etc.). Thus, in-depth analyses of the numerous roles of different types of helicases/topoisomerases in stress adaptation would prove beneficial for developing stress tolerant crops in future.

## Author contributions

SS and MVR conceived the experiments. RJ, UG, and BS performed the experiments and generated data. MKR provided the gene construct. MVR, SS, and TK analyzed the data, and corrected and edited the manuscript. All the authors approved the final manuscript.

### Conflict of interest statement

The authors declare that the research was conducted in the absence of any commercial or financial relationships that could be construed as a potential conflict of interest.
